# Clinical Value of RNA Sequencing–Based Classifiers for Prediction of the Five Conventional Breast Cancer Biomarkers: A Report From the Population-Based Multicenter Sweden Cancerome Analysis Network—Breast Initiative 

**DOI:** 10.1200/PO.17.00135

**Published:** 2018-03-09

**Authors:** Christian Brueffer, Johan Vallon-Christersson, Dorthe Grabau†, Anna Ehinger, Jari Häkkinen, Cecilia Hegardt, Janne Malina, Yilun Chen, Pär-Ola Bendahl, Jonas Manjer, Martin Malmberg, Christer Larsson, Niklas Loman, Lisa Rydén, Åke Borg, Lao H. Saal

**Affiliations:** **Christian Brueffer**, **Johan Vallon-Christersson**, **Anna Ehinger**, **Jari Häkkinen**, **Cecilia Hegardt**, **Yilun Chen**, **Pär-Ola Bendahl**, **Jonas Manjer, Christer Larsson**, **Niklas Loman**, **Lisa Rydén**, **Åke Borg**, and **Lao H. Saal**, Lund University, Lund; **Dorthe Grabau**, **Anna Ehinger**, **Martin Malmberg**, **Niklas Loman**, and **Lisa Rydén**, Skåne University Hospital Lund, Lund; **Anna Ehinger**, Blekinge County Hospital, Karlskrona; and **Janne Malina** and **Jonas Manjer**, Skåne University Hospital Malmö, Malmö, Sweden.

## Abstract

**Purpose:**

In early breast cancer (BC), five conventional biomarkers—estrogen receptor (ER), progesterone receptor (PgR), human epidermal growth factor receptor 2 (HER2), Ki67, and Nottingham histologic grade (NHG)—are used to determine prognosis and treatment. We aimed to develop classifiers for these biomarkers that were based on tumor mRNA sequencing (RNA-seq), compare classification performance, and test whether such predictors could add value for risk stratification.

**Methods:**

In total, 3,678 patients with BC were studied. For 405 tumors, a comprehensive multi-rater histopathologic evaluation was performed. Using RNA-seq data, single-gene classifiers and multigene classifiers (MGCs) were trained on consensus histopathology labels. Trained classifiers were tested on a prospective population-based series of 3,273 BCs that included a median follow-up of 52 months (Sweden Cancerome Analysis Network—Breast [SCAN-B], ClinicalTrials.gov identifier: NCT02306096), and results were evaluated by agreement statistics and Kaplan-Meier and Cox survival analyses.

**Results:**

Pathologist concordance was high for ER, PgR, and HER2 (average κ, 0.920, 0.891, and 0.899, respectively) but moderate for Ki67 and NHG (average κ, 0.734 and 0.581). Concordance between RNA-seq classifiers and histopathology for the independent cohort of 3,273 was similar to interpathologist concordance. Patients with discordant classifications, predicted as hormone responsive by histopathology but non–hormone responsive by MGC, had significantly inferior overall survival compared with patients who had concordant results. This extended to patients who received no adjuvant therapy (hazard ratio [HR], 3.19; 95% CI, 1.19 to 8.57), or endocrine therapy alone (HR, 2.64; 95% CI, 1.55 to 4.51). For cases identified as hormone responsive by histopathology and who received endocrine therapy alone, the MGC hormone-responsive classifier remained significant after multivariable adjustment (HR, 2.45; 95% CI, 1.39 to 4.34).

**Conclusion:**

Classification error rates for RNA-seq–based classifiers for the five key BC biomarkers generally were equivalent to conventional histopathology. However, RNA-seq classifiers provided added clinical value in particular for tumors determined by histopathology to be hormone responsive but by RNA-seq to be hormone insensitive.

## INTRODUCTION

Histopathologic analysis of breast cancers (BCs) for estrogen receptor (ER) and progesterone receptor (PgR) content, human epidermal growth factor receptor 2 (HER2) gene amplification, and Nottingham histologic grade (NHG) are the mainstays of current clinical practice.^1^ Increasingly, assessment of the proliferation antigen Ki67 is clinically recommended.^2^ These five biomarkers carry prognostic and predictive information and are used in combination with other clinicopathological factors for risk stratification and therapy selection.^1^

Current evaluation of these BC biomarkers is imperfect. Immunohistochemistry (IHC) is the principal method for ER, PgR, HER2, and Ki67 measurement, and in situ hybridization (ISH) methods are used to refine HER2 IHC. Among laboratories, significant differences exist in, for example, fixation, antigen retrieval, antibodies, chemistries, scoring systems, and interpretation. Accuracy and reproducibility are concerns, with up to 20% false-positive or false-negative ER/PgR IHC determinations.^3^ Varying discordance has been reported for HER2 IHC and fluorescent ISH (FISH).^4-7^ Accordingly, consensus guidelines emphasize standardization and validation of analytic performance.^1,2,8^ Lack of standardization has slowed the entrance of Ki67 into clinical routines.^9^ For example, Ki67 status was only moderately concordant in an interlaboratory reproducibility analysis.^10^ Thresholds for Ki67 positivity are evolving; cutoffs between 20% and 29% were recommended by the 2015 St Gallen/Vienna panel for laboratories with a quality assurance program.^11^ Swedish quality assurance program guidelines recommend that each laboratory calibrate a cutoff yearly such that one third of 100 consecutive occurrences are Ki67-high. The NHG system was developed to establish better standards and improve reproducibility, and it is the recommended method for BC grading today. NHG reproducibility studies^12^ have reported modest agreements (pairwise κ, 0.43 to 0.83), which correspond to 15% to 30% discordance.

Microarray and reverse transcriptase polymerase chain reaction–based gene expression analyses of BCs have yielded many signatures for tumor subtyping, prognosis, and survival, as well as for individual biomarkers, such as ER, PgR, HER2, and PTEN.^13-16^ Massively parallel sequencing of mRNA (RNA-seq) has advantages compared with earlier methods, including greater dynamic range and reproducibility and the ability to discover and quantify transcripts without a priori sequence knowledge. In 2010, toward implementation of molecular profiling in the clinical routine, we launched the Sweden Cancerome Analysis Network Breast Initiative (SCAN-B; ClinicalTrials.gov identifier: NCT02306096), an ongoing population-based multicenter observational study covering a wide geography of Sweden that prospectively invites all patients with BC to participate.^17^ To date, approximately 85% of the eligible catchment population are included, more than 11,000 patients have enrolled, and blood and fresh tumor tissues are sampled for molecular research. In the first phase, all tumors are analyzed by RNA-seq generally within 1 week after surgery. Thus, for each BC, it will be possible to report a multitude of biomarker tests simultaneously on the basis of its RNA-sequencing data and within a clinically actionable time frame.

Herein, we aimed to validate the SCAN-B multicenter infrastructure and provide molecular analyses of clinical value by developing RNA-seq–derived classifiers for the conventional histopathologic BC biomarkers ER, PgR, HER2, Ki67, and NHG. For this purpose, both single-gene classifiers (SGCs) and multigene classifiers (MGCs) were developed by using a training cohort, the prediction accuracy was compared against current clinical practice across a large independent prospective cohort, and the classifier predictions and their discrepancies to histopathology were evaluated with respect to patient survival.

## METHODS

### Patients

**Fig 1.**
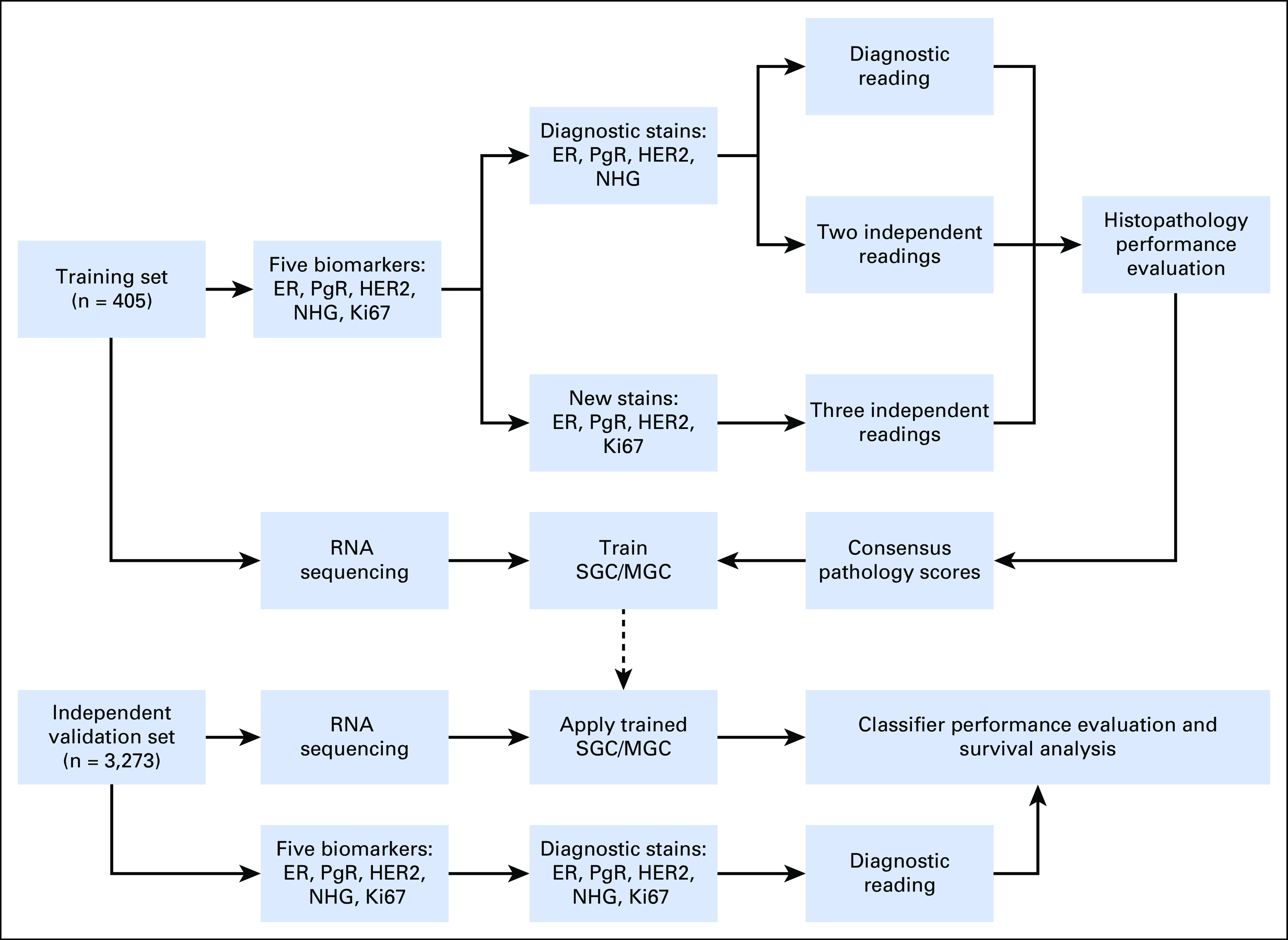
Study design flow diagram. ER, estrogen receptor; HER2, human epidermal growth factor receptor 2; Ki67, proliferation antigen Ki67; MGC, multigene classifier; NHG, Nottingham histologic grade; PgR, progesterone receptor; SGC, single-gene classifier.

**Fig A1.**
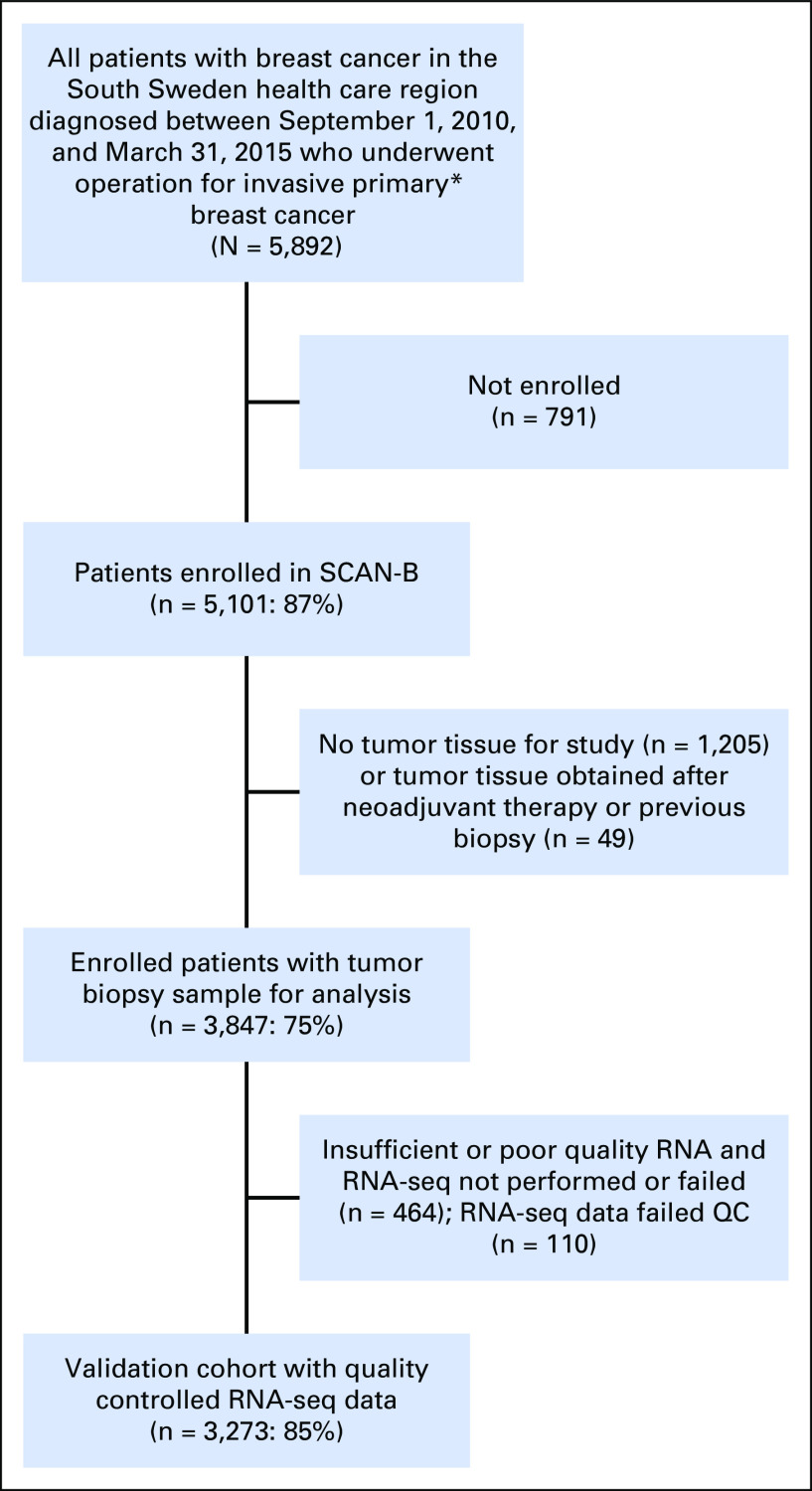
Flow diagram for Sweden Cancerome Analysis Network—Breast (SCAN-B) population-based 3,273-tumor independent validation cohort. (*) Nonmetastatic primary unilateral breast cancer, which excluded patient cases that had a diagnosis of synchronous (< 3 months) contralateral invasive breast cancer. QC, quality control.

The study (Fig 1) was approved by the Regional Ethical Review Board of Lund at Lund University and the Swedish Data Inspection group. Health professionals provided patient information, and patients gave written informed consent. Clinical data were retrieved from the Swedish National Breast Cancer Registry. Diagnostic pathology slides, snap-frozen surgical tumor specimens, and formalin-fixed paraffin-embedded tissue blocks were retrieved for 405 patient cases, selected for classifier training with an over-representation of HER2-positive and ER-negative tumors (training cohort; Data Supplement). For classifier testing, an independent, prospective, and population-based modern cohort of 3,273 patients with early BC was assembled from the ongoing SCAN-B study^17^ (validation cohort; Appendix Fig A1; Data Supplement).

### Histopathology

For the training cohort, all biomarkers with the exception of Ki67 were evaluated at time of diagnosis. In addition, new formalin-fixed paraffin-embedded slides were analyzed for ER, PgR, and Ki67 IHC and for HER2 silver ISH, all performed at a central laboratory (Helsingborg Hospital). The diagnostic slides and newly stained slides were each scored in total by three pathologists independently by using 1% or greater tumor cell staining threshold for hormone receptor positivity, standard HER2 HercepTest (Agilent/Dako, Santa Clara, CA) and ISH criteria (Roche/Ventana, Tucson, AZ), greater than 20% positive nuclei for Ki67-high status, and the NHG scoring system (Data Supplement). On the basis of all evaluations, a consensus score for each biomarker was determined with the majority scores.

### Tumor Processing and RNA Sequencing

Snap-frozen (training cohort) or RNAlater-preserved (validation cohort) tumor specimens were processed and sequenced, and the raw data (Data Supplement) was processed as described previously.^17,18^ All data are available from the NCBI Gene Expression Omnibus (Accession Nos. GSE81538 and GSE96058).

### Classifiers

Within the 405-patient training set, SGCs were built for the ER, PgR, HER2, and Ki67 biomarkers by determining the optimal expression thresholds for the genes *ESR1*, *PGR*, *ERBB2*, and *MKI67* that maximized concordance to the respective histopathology consensus score (Data Supplement). MGCs for ER, PgR, HER2, Ki67, and NHG were built by training nearest shrunken centroid (NSC)^19^ models with the 5,000 most varying genes across the training cohort (Data Supplement) and the histopathology consensus scores as training labels. Within the training set, optimal model parameters were determined by using cross-validation and then were used to train prediction models with all training samples. The resulting four SGCs and five MGCs were used to predict the biomarker status of 3,273 independent validation BC samples. The biologic functional annotation clusters of each MGC signature were evaluated with the DAVID Bioinformatics Resource.^20^

### Statistical Analysis

Histopathology evaluations and single-gene and multigene predictions were compared with agreement statistics^21^ (defined in the Data Supplement) and balanced statistics—Cohen’s κ and Matthews correlation coefficient (MCC)—and were interpreted according to Viera and Garrett.^22^ The κ and MCC values were comparable (Data Supplement), so we focused on κ. Kaplan-Meier and Cox regression survival analyses were performed with overall survival as the end point. Multivariable Cox models included the variables age at diagnosis, lymph node status, tumor size, ER, PgR, HER2, and NHG as covariates, as relevant (Data Supplement). All calculations were performed with R 3.2.3. *P* values of ≤ .05 were considered significant.

## RESULTS

### Clinical Histopathology

To estimate the inherent variability within clinical histopathology and to determine a consensus score for each BC biomarker for classifier training, a comprehensive histopathologic analysis was performed for 405 patient breast tumors with three readings of up to two independent stains for the five conventional biomarkers: ER, PgR, HER2, Ki67, and NHG (Fig 1). With the diagnostic evaluation as the reference, agreement statistics were calculated (Table 1; Data Supplement). Concordance for histopathologic evaluation of ER, PgR, and HER2 into positive and negative groups was high; the average pairwise agreements were 97.3% (average κ [Aκ], 0.920), 95.5% (Aκ, 0.891), and 96.6% (Aκ, 0.899), respectively, whereas agreements were lower for Ki67 (86.8%; Aκ, 0.734) and NHG (74.8%; Aκ, 0.581). As expected with minimization of technical and heterogeneity factors, within-slide concordances were slightly better than between-slide concordances (Data Supplement).

**Table 1.**
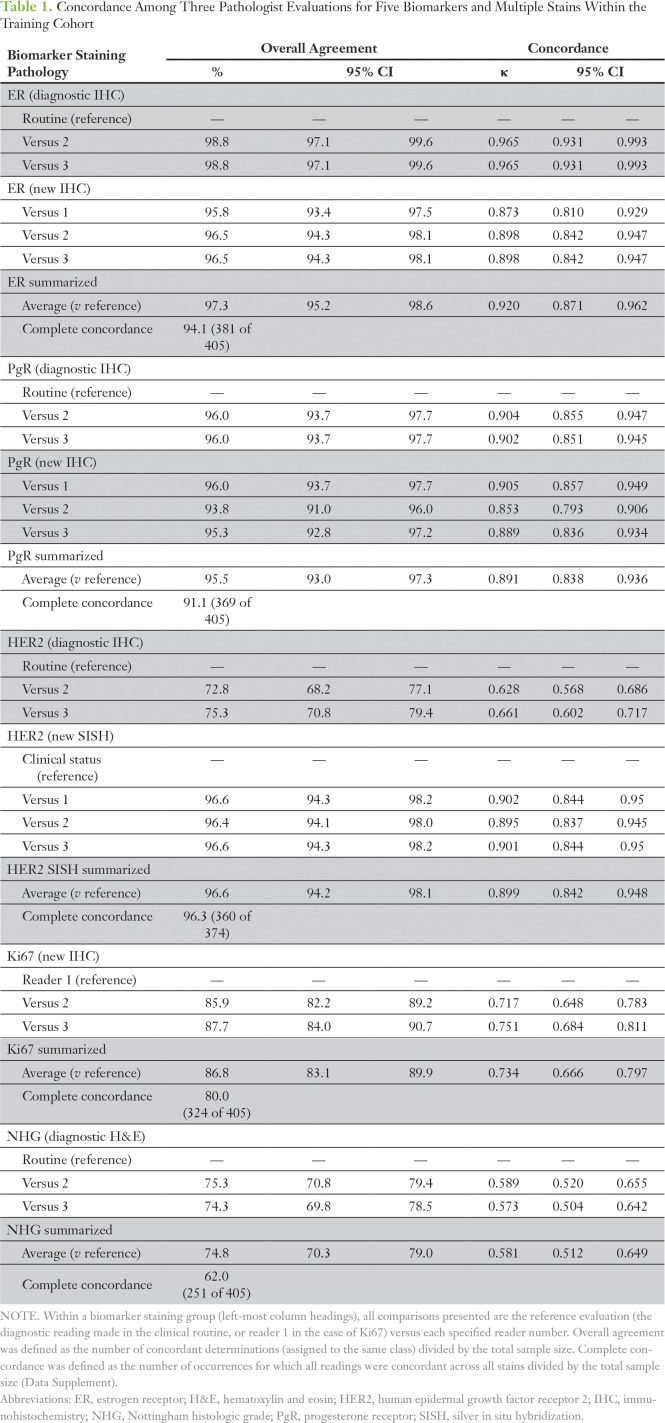
Concordance Among Three Pathologist Evaluations for Five Biomarkers and Multiple Stains Within the Training Cohort

### Classifier Training

Whole-transcriptome expression profiles were generated for the 405 training samples using RNA-seq. For the SGCs, optimal thresholds were determined for *ESR1* (which encodes the ER protein), *PGR* (PgR), *ERBB2* (HER2), and *MKI67* (Ki67) (Data Supplement). Next, MGCs were trained, and the training-cohort cross-validation accuracy was determined (balanced accuracy or accuracy ± standard deviation; Data Supplement) as follows: ER, 95.3% ± 2.4%; PgR, 90.4% ± 2.9%; HER2, 88.5% ± 3.8%; Ki67, 84.9% ± 3.4%; and NHG, 73.8% ± 3.9%. For MGCs, the NSC method has the property of eliminating noninformative genes (zero weight for the classification). The ER classifier had 459 weighted genes; PgR, 184; HER2, 312; Ki67, 273; and NHG, 206 (Data Supplement). In total, 869 genes had nonzero weights in at least one MGC classifier. The constituent biologic themes for each MGC classifier were investigated with functional annotation clustering (Data Supplement).

### Performance on Independent Data

**Fig 2.**
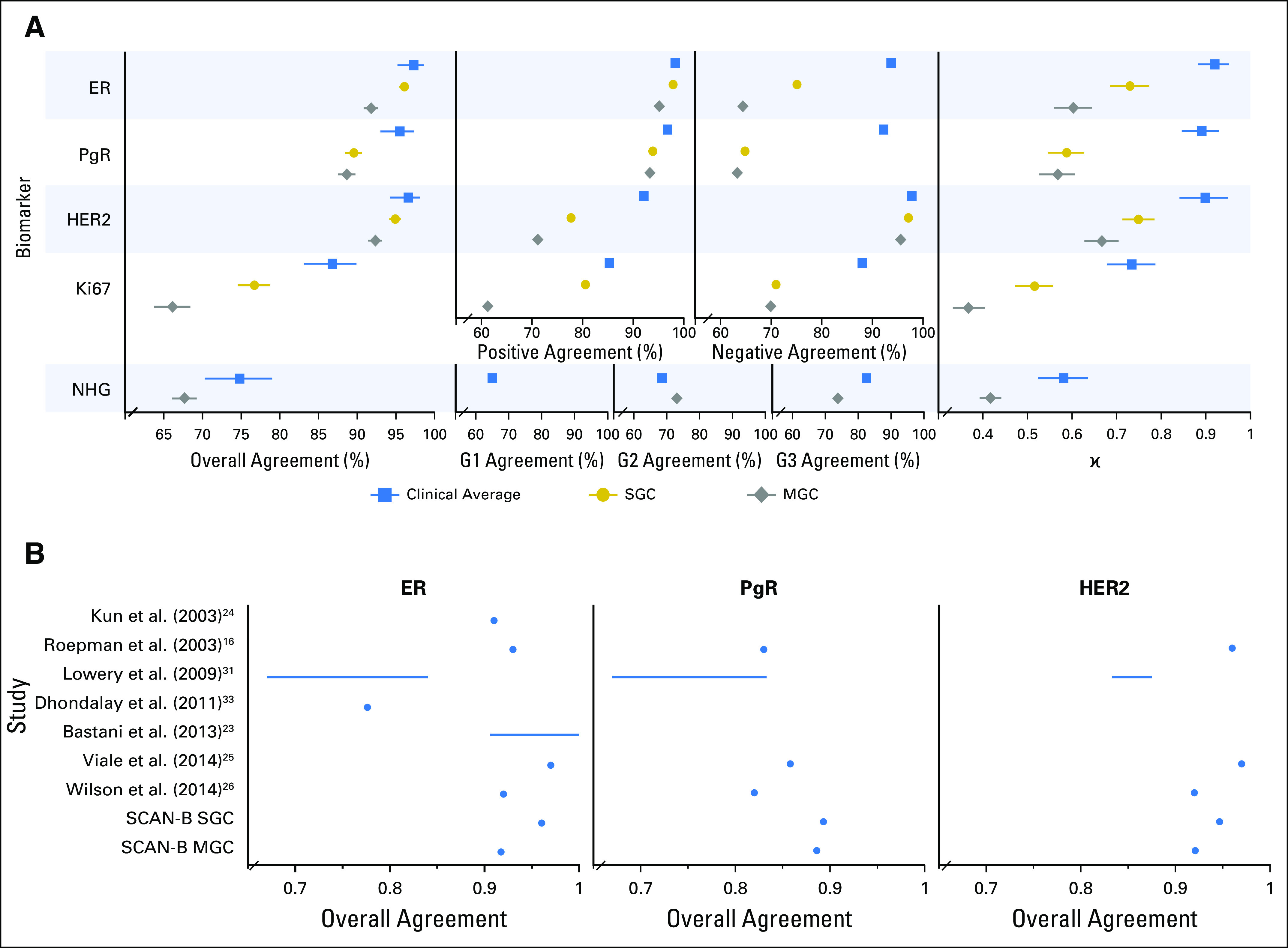
Performance of trained classifiers in the 3,273-tumor independent validation cohort. (A) Forest plots of concordance statistics for histopathologic evaluation in the training set (blue square markers), and single-gene classifiers (SGCs; gold circles) and multigene classifiers (MGCs; gray diamonds) in the validation cohort, which plots overall agreement with 95% CIs, specific agreements (positive and negative agreements for estrogen receptor [ER], progesterone receptor [PgR], human epidermal growth factor receptor 2 [HER2], and Ki67) and Nottingham histologic grade (NHG) category agreements (grade [G] 1, G2, and G3), and κ values with 95% CIs. Overall agreement is defined as the number of concordant determinations (assigned to the same class) divided by the total sample size. Positive, negative, and G1/G2/G3 agreements are the proportions of agreement specific to the given category (Data Supplement). (B) Overall agreement of classifiers from the literature compared with our SGCs and MGCs. SCAN-B, Sweden Cancerome Analysis Network—Breast.

**Fig A2.**
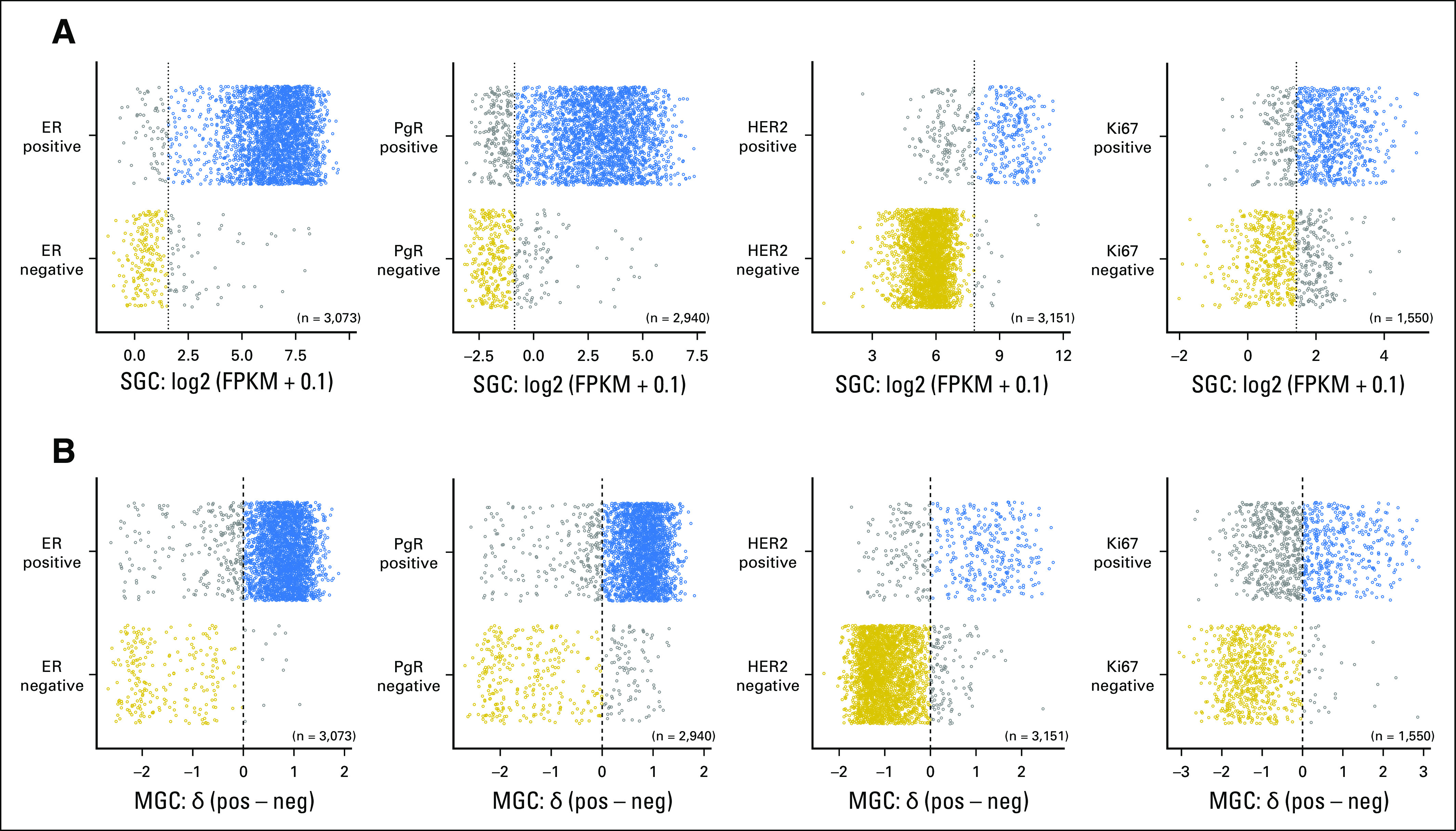
Prediction of biomarker status in the 3,273-case independent validation cohort. For estrogen receptor (ER), progesterone receptor (PgR), human epidermal growth factor receptor 2 (HER2), and Ki67 clinical histopathology diagnostic results (y-axis), the single-gene classifier (SGC) gene expression (x-axis) (A) or the transformed multigene classifier (MGC) score (x-axis) (B) is plotted for the validation cohort (circles). Within a biomarker prediction, gold circles were concordantly biomarker negative, blue circles were concordantly positive, and gray circles were discordant by the classifier or histopathology. Vertical dotted (SGC) and dashed (MGC) lines represent the classifier threshold that distinguished the classes. FPKM, fragments per kilobase of transcript per million mapped reads.

**Fig A3.**
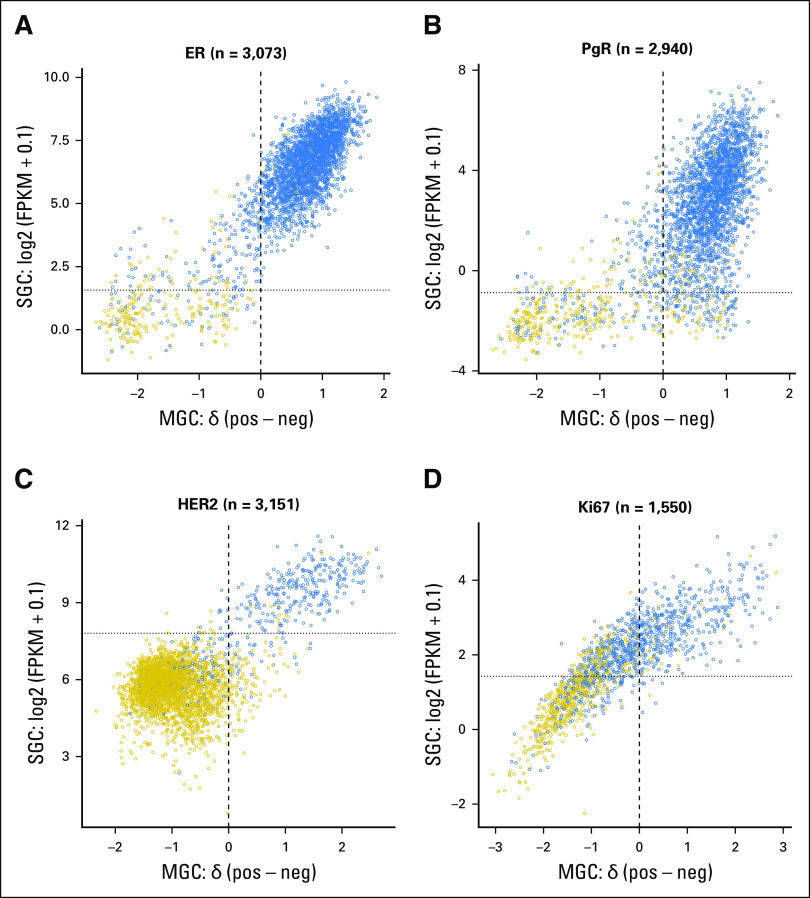
Transformed multigene classifier (MGC) score (x-axis) versus single-gene classifier (SGC) gene expression (y-axis) in the 3,273 samples of the independent validation cohort (circles) for (A) estrogen receptor (ER), (B) progesterone receptor (PgR), (C) human epidermal growth factor receptor 2 (HER2), and (D) Ki67. Gold circles are negative or low by histopathology, and blue circles are positive or high by histopathology. Vertical dashed lines are drawn at the MGC score threshold of 0 to distinguish the classes, and horizontal dotted lines are drawn at the SGC gene expression thresholds determined from the training cohort. FPKM, fragments per kilobase of transcript per million mapped reads.

To evaluate the classifiers, we tested them on RNA-seq data generated for 3,273 independent tumors from the prospective population-based multicenter SCAN-B study (n = 136 tumors were analyzed in technical replicates). Concordance between the diagnostic histopathologic results and the SGC predictions was substantial for ER (overall agreement, [OA], 96.1%; κ, 0.730) and HER2 (OA, 94.92%; κ, 0.749) and moderate for PgR (OA, 89.6%; κ, 0.588) and Ki67 (OA, 76.7%; κ, 0.516; Fig 2; Appendix Figs A2 and A3; Data Supplement). Similarly, for the MGCs, concordance was substantial for ER (OA, 91.9%; κ, 0.606) and HER2 (OA, 92.4%; κ, 0.667), moderate for PgR (OA, 88.7%; κ, 0.568) and NHG (OA, 67.7%; κ, 0.418), and fair for Ki67 (OA, 66.3%; κ, 0.370). For RNA-seq replicates, 534 (98.2%) of 544 SGC classifications and 675 (99.3%) of 680 MGC classifications were concordant (Data Supplement). Similar results were obtained when an ER/PgR IHC cutoff of 10% or greater positive cells (current Swedish standard) was used.

### Survival Analysis

**Fig 3.**
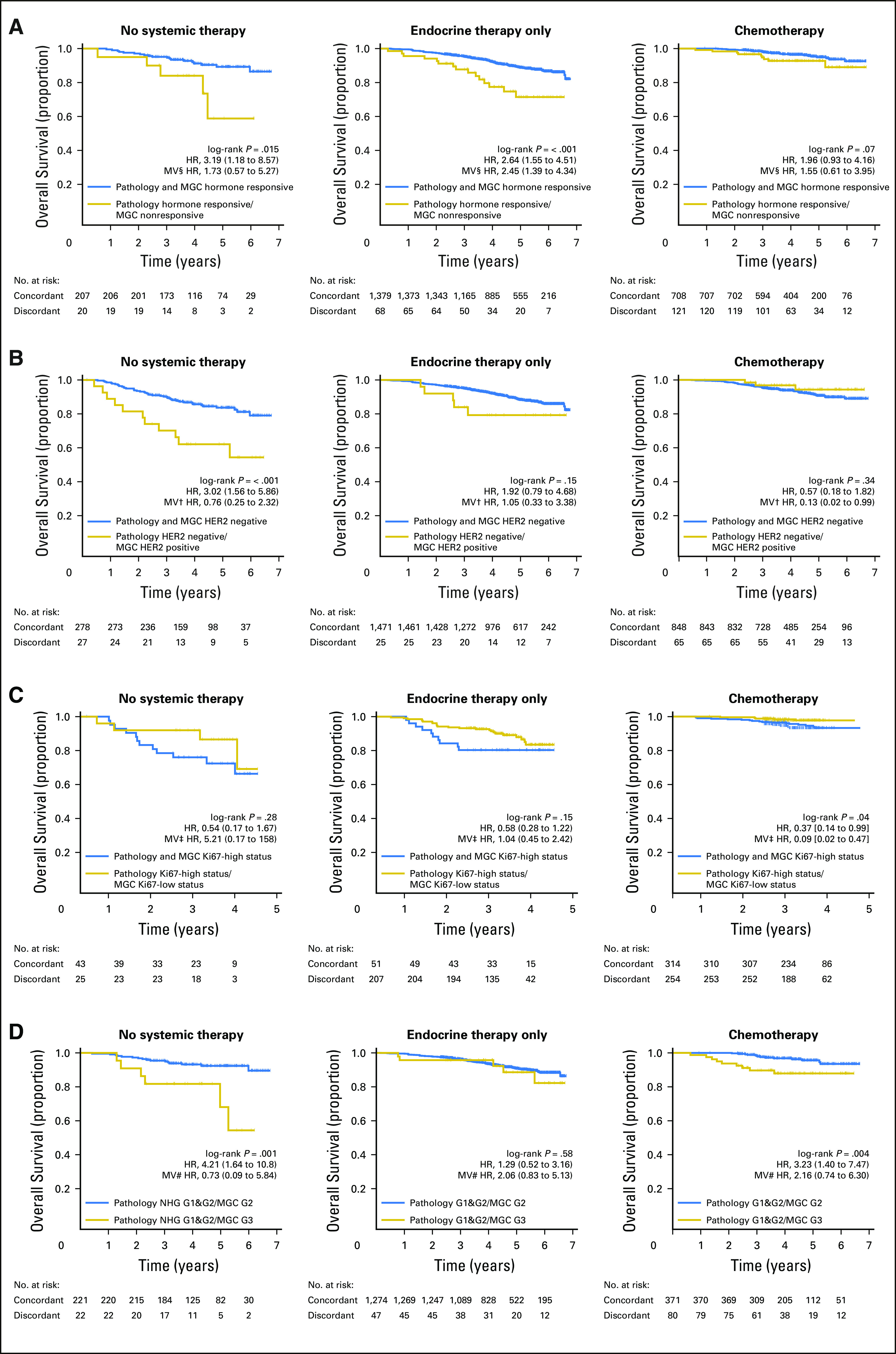
Kaplan-Meier overall survival estimates and Cox regression survival analysis for multigene classifiers (MGCs) within the independent validation cohort. (A) Histopathologically hormone responsive (defined as estrogen receptor [ER] positive and progesterone receptor [PgR] positive) group stratified by MGC hormone responsive classification (concordant [blue curve] or discordant [gold curve] to histopathology) within the subgroup of patients who received (left) no adjuvant systemic therapy, (middle) endocrine therapy alone, or (right) chemotherapy with or without trastuzumab or endocrine therapy. (B) Human epidermal growth factor receptor 2 [HER2]–negative histopathology group stratified by HER2 MGC for the same three treatment subgroups as in A. (C) Ki67-high histopathology group stratified by Ki67 MGC for the same three treatment subgroups as in A. (D) Nottingham histologic grade (NHG) combined grade [G] 1 and G2 histopathology group stratified by NHG MGC for the same three treatment subgroups as in A. In each Kaplan-Meier plot, the histopathology to MGC concordant tumor cases are plotted in blue, the discordant tumor cases are plotted in gold, the log-rank *P* value is given, and the hazard ratio (HR) for discordant-versus-concordant result is given with a 95% CI and after multivariable (MV) Cox regression adjustment. Covariables included in the MV analysis were age at diagnosis, lymph node status, tumor size, and the variables denoted by the following symbols: †, ER, PgR, and NHG; ‡, ER, PgR, HER2, and NHG; §, HER2 and NHG; #, ER, PgR, and HER2.

**Fig A4.**
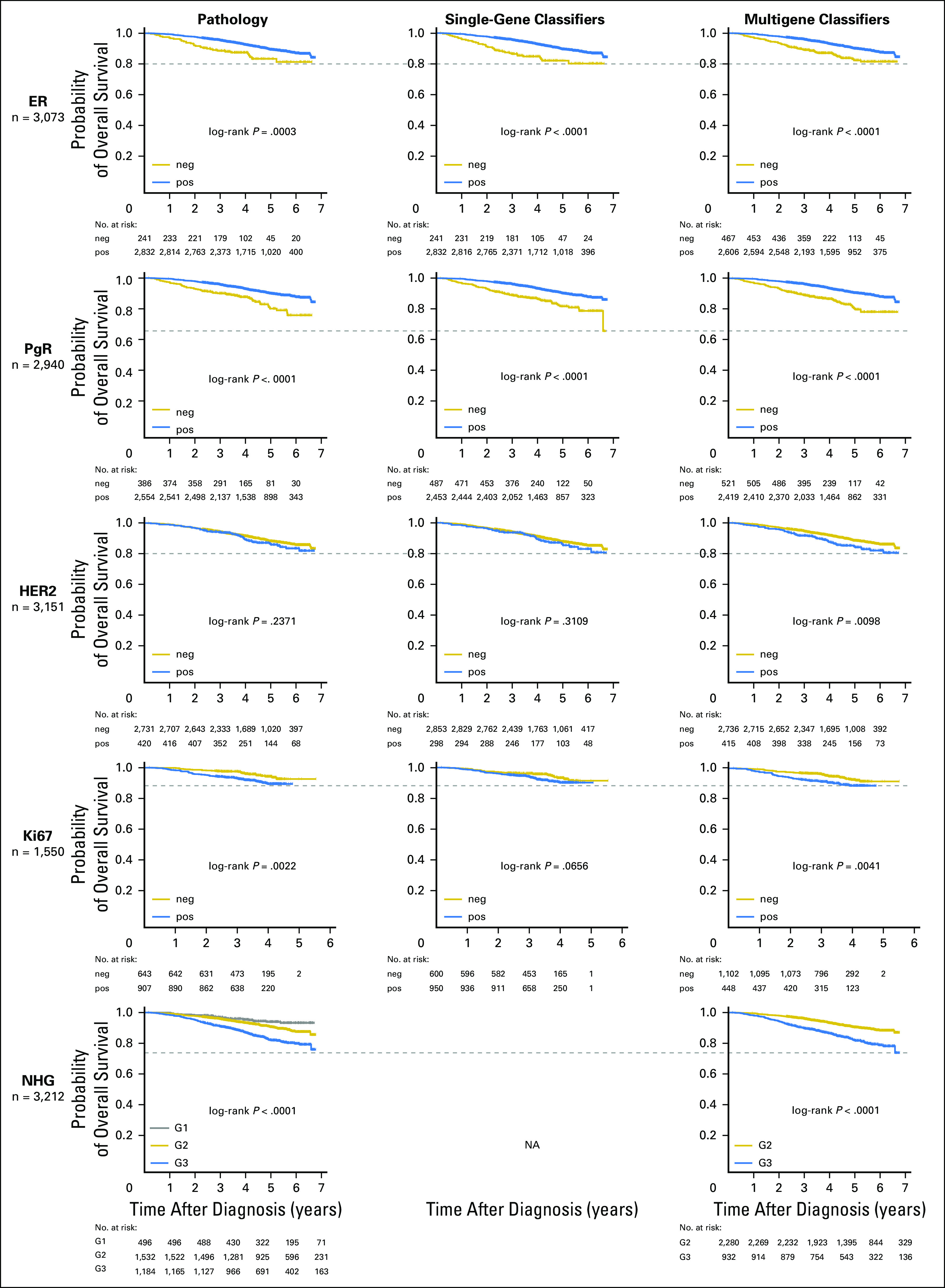
Kaplan-Meier overall survival estimates for histopathology, single-gene classifiers (SGCs), and multigene classifiers (MGCs) within the validation cohort (neg, classified as negative; pos, classified as positive; grade [G]1, G2, or G3). The biomarker is indicated at the far left, and the number of tumor cases with complete data across pathology, SGC, and MGC for a given biomarker is shown below each biomarker name. In columns are plotted the Kaplan-Meier survival curves for each classification: (left) pathology, (middle) SGC, and (right column) MGC. The log-rank *P* value is displayed, and horizontal dashed lines are drawn to aid identification of Kaplan-Meier estimates with the poorest outcome classification group within each row. Generally, histopathology and SGCs had similar curves, whereas the MGCs had noticeably improved stratification, for the hormone receptors, in particular.

**Fig A5.**
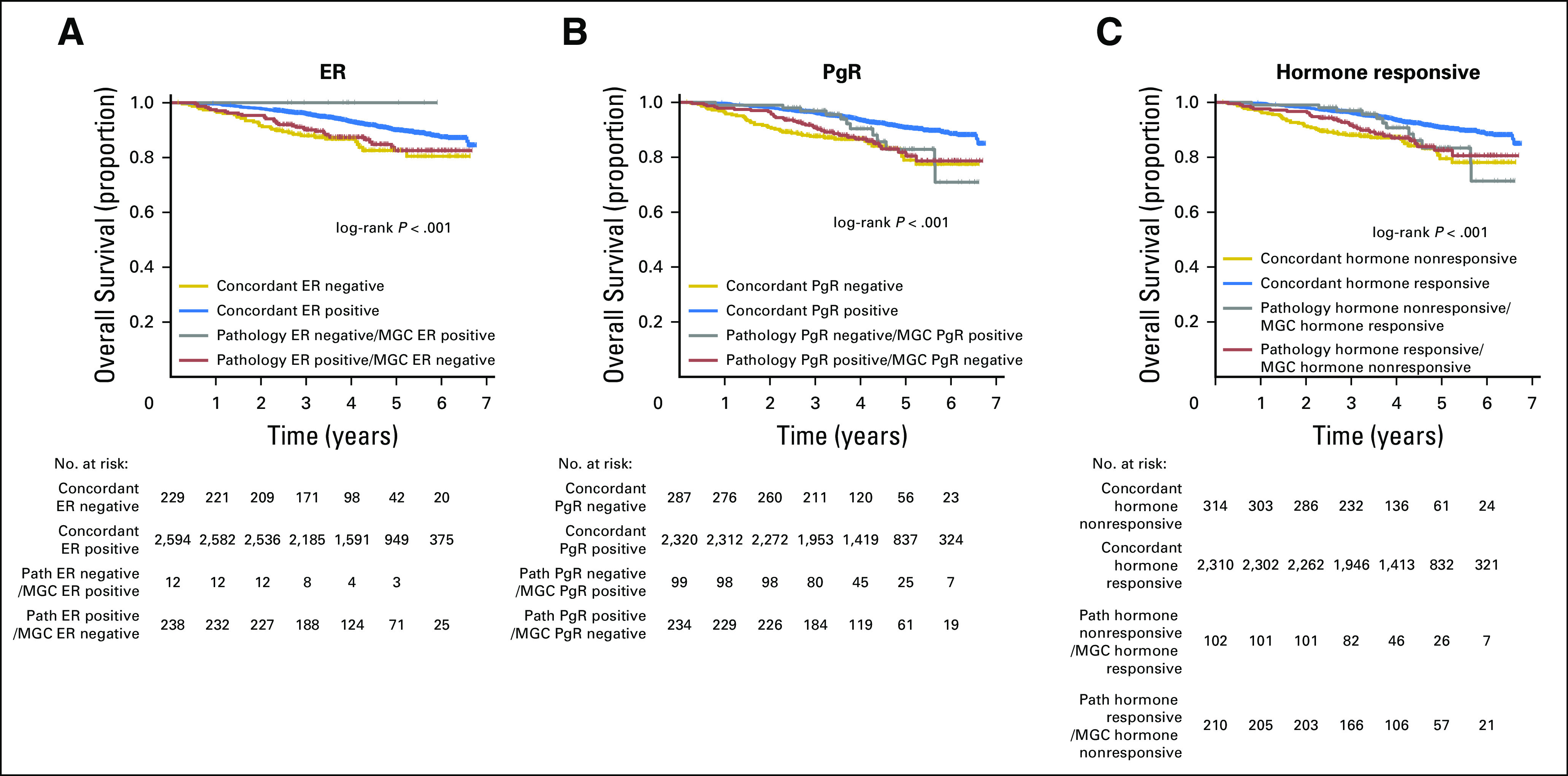
Kaplan-Meier overall survival estimates for groups defined by pathology (path) versus multigene classifiers (MGCs) within the validation cohort; the log-rank *P* value is given. (A) The entire validation cohort stratified by concordance or discordance between estrogen receptor (ER) histopathology and the ER MGC. (B) Progesterone receptor (PgR) status stratified by histopathology and PgR MGC. (C) Hormone responsiveness status stratified by histopathology and MGC; responsive is defined as ER and PgR positive; nonresponsive, as ER negative or PgR negative.

To evaluate the possible clinical utility of our classifiers, we analyzed our classifier predictions within the validation cohort with respect to overall survival. Kaplan-Meier analysis revealed comparable patient stratification for both diagnostic histopathology and SGCs for the five biomarkers across the entire validation cohort, whereas the MGCs had a noticeably richer stratification, particularly for the hormone receptors and the hormone-responsive group, defined by ER positivity and PgR positivity (Appendix Figs A4 and A5). Therefore, and to reduce the number of comparisons, we focused on the MGCs for each biomarker and within the major treatment groups. Patients with tumors discrepant for hormone responsiveness (hormone responsive by pathology but not responsive by MGC) had significantly worse outcomes across the entire cohort (hazard ratio [HR], 1.64; 95% CI, 1.17 to 2.28; log-rank *P* = .0034) as well as within subgroups defined by adjuvant treatment: no systemic therapy (HR, 3.19; 95% CI, 1.19 to 8.57; *P* = .015) and only endocrine therapy (HR, 2.64; 95% CI, 1.55 to 4.51; *P* < .001; Fig. 3A). Furthermore, MGC predictions added value to predictions of HER2, Ki67, and NHG (Figs 3B to 3D). After adjusting for important covariates in multivariable Cox analyses, the MGC prediction for hormone nonresponsiveness was a significant stratifier among patients with histopathologic hormone-responsive disease who were treated with endocrine therapy, as were the MGC predictions discordant for HER2-negative or Ki67-high status in patients who received chemotherapy with or without trastuzumab and/or endocrine therapy. Conversely, the NHG MGC became nonsignificant (Fig 3).

## DISCUSSION

Despite efforts to develop better standards for clinical histopathologic evaluation of breast tumors, intra/interlaboratory and -reader variation remain problematic. Previously, several gene expression–based approaches for determination of known treatment-predictive biomarkers have been developed^16,23-26^; however, they are not widely used clinically in most countries. Supplementation of histopathologic biomarkers with biomarkers determined from RNA-seq profiling is becoming feasible today: costs are less than $300 per transcriptome, and projects, such as SCAN-B and others, that use RNA-seq in the clinic are emerging.^17,27,28^ In this study, we demonstrated that accurate classifiers for ER, PgR, HER2, Ki67 and NHG can be built with RNA-seq data, can provide a valuable complement to traditional histopathology, and represent the first of many potential clinical reports that can be delivered from a single RNA-seq measurement. In the future, we foresee the development, validation, and clinical implementation of a multitude of signatures, classifiers, and mutational profiles within the SCAN-B population-based infrastructure and RNA-seq platform.^17,18^ We also aim to use RNA-seq analyses in the performance of interventional clinical trials.^29^

The quality of machine-learned classifiers is crucially dependent on the quality of the labels on which they have been trained. To ensure highly accurate pathology labels, we sought to reduce variance by generating consensus scores for each biomarker. Matched against routine histopathologic evaluation, repeated ER, PgR, and HER2 readings showed good concordance, whereas Ki67 and NHG had notably lower concordance between pathologists (Table 1). Reproducibility of tumor grading systems has long been debated,^30^ and Ki67 has been shown to have high intralaboratory but low interlaboratory reproducibility.^10^ Here, the histopathologic variability was highest for Ki67 and NHG, which added uncertainty even to our consensus scores. It is unlikely that a classifier would perform better than the quality of training labels; therefore, it is not surprising that our classifiers had the worst performance for Ki67 and NHG. Moreover, because we benchmarked our biomarker predictions in the validation cohort to the clinical diagnostic histopathology results that contained this inherent variability, we could not expect our classifiers to have higher accuracy than what is achievable within histopathology.

Generally, SGCs performed comparably to clinical diagnostic pathology. The SGC ER and HER2 classifiers had substantial κ agreement compared with the clinical average, and PgR and Ki67 had moderate agreement. Likewise, our MGCs had comparable performance. The MGC ER and HER2 classifiers had substantial agreement in line with the clinical average, whereas PgR and NHG classifiers had moderate agreement, and the Ki67 classifier had fair agreement. Earlier work on mRNA-based classifiers for ER, PgR, and HER2 has been performed with microarrays, quantitative reverse-transcriptase polymerase chain reaction, and, recently, with RNA-seq and mainly has been restricted to signatures of either one^16,31^ or few^23,24,26,32,33^ genes. The performance of our classifiers generally were in line with the results of these previous studies, which indicates the suitability of our RNA-seq approach (Fig 2B).

Discrepancies between RNA-seq–based classifications and histopathology may be a result of staining and reader variations, as discussed in this paper. Discrepancies may also develop from tissue sampling and heterogeneity, in which the specimen used for sequencing may not be representative of the piece selected for histopathology. Another consideration is the biologic layer at which biomarker status is assessed: mRNA versus protein abundance or DNA copy number. The consequence is that a mismatch between mRNA biomarker prediction and histopathology may be influenced by various mechanisms active between these layers, for example RNA silencing/interference/translation, protein stability and epitope availability, or tumor heterogeneity.

Despite these possible explanations for discrepancies, when benchmarked against patient outcome, our classifiers exemplified enhanced stratification of patients with significant differences in overall survival (Fig 3; Appendix Figs A4 and A5). The fact that MGCs performed best overall suggests that a multigene signature captures the biologic signaling up- and down-stream of the biomarker in question in a more consequential way than the expression of the single gene or protein alone. This conclusion is supported by each signature’s underlying biologic themes and pathways (Data Supplement), and by our observation for technical replicates, in which MGCs had near-perfect reproducibility and an error rate that was approximately half that of SGCs (0.7% *v* 1.8%). Ultimately, these results can be used to identify patients who may benefit from additional treatment. Another approach is to use clinical outcome as the training labels to develop new prognostic/predictive signatures.^13,34^ The SCAN-B material is excellently suited to evaluate previously published signatures; as we accrue longer follow-up, we aim to develop RNA-seq signatures trained on clinical outcomes.

Ki67 has been introduced relatively recently in international guidelines.^11^ To our knowledge, this study is the first to develop a validated predictor for Ki67 status. The lower concordance between our Ki67 predictions compared with the clinical reference is related to the relatively larger Ki67 interrater disagreement seen within our consensus pathology evaluation, which is likely a consequence of the continuous nature of Ki67 expression and of the spectrum of proliferation activity and pathways in BC.

NHG is distinct from the other biomarkers. It has no single underlying gene but rather is a compound biomarker that consists of three morphologic properties: tubular differentiation, nuclear pleomorphism, and mitotic count. Moreover, NHG prediction is a three-class problem. Even for pathologists, NHG can be difficult to determine, as evidenced by the moderate κ and OA results within clinical pathology, in line with the literature.^12^ Most misclassified tumor cases in this study were histologically grade 1 (G1) or grade 3 that were misclassified as grade 2 (G2) by our predictor. Large interrater disagreement, especially for G1 and G2, could explain the results of our classifier with only moderate OA to histopathology (67.7%). All histologic G1 occurrences were misclassified, which may have been a result of the imbalanced composition of the training set for NHG (48 of 405 samples consensus-scored G1), or may have occurred because G1 is not a discrete entity but rather the lower end of an underlying continuous scale. Indeed, Kaplan-Meier analysis showed that the curves G1 and G2 largely overlapped in the validation cohort (Appendix Fig A4). Another approach, instead of recapitulation of the pathology grading scheme, could be to reduce the problem to a binary classification of either low or high grade. This approach has been suggested by others as a viable gene-expression–based alternative to NHG for translation into a clinical setting^35,36^ and essentially is what our NHG predictor has become.

An important question when building classifiers is how many genes to use. We compared single-gene and multigene classifiers. When compared with clinical pathology, SGCs have slightly better concordance than MGCs for ER and HER2, whereas the SGC and MGC performances were comparable for PgR and Ki67. This difference may have developed because these biomarkers are faithfully represented by their associated single genes. Another consideration for classifiers is robustness toward missing values. MGCs may be more robust than SGCs, because they are able to classify tumors correctly even when the main gene that underlies a biomarker is poorly measured in a particular analysis. When clinical outcome was considered, the survival analyses indicated that our MGCs generally contained greater potential clinical utility than SGCs to complement histopathology.

In summary, we have performed a systematic pathologic evaluation of 405 BC tumors, which resulted in consensus scores for the five conventional BC biomarkers and estimated a well-controlled best-case scenario for the inherent uncertainty within clinical histopathology. With tumor RNA-seq data and the consensus scores, we trained SGCs and MGCs and evaluated the classifiers on an independent set of 3,273 tumors. The accuracy of our classifiers was comparable to the inherent accuracy of clinical pathology and was highly reproducible. Classifiers based on the expression of single genes performed slightly better than MGCs for concordance to histopathology, but MGCs performed significantly better for stratification of patients into groups with clinically meaningful differences in survival, in particular for histopathologic hormone-responsive BCs. In conclusion, RNA-seq–based classifiers may be suitable complementary diagnostics for BC, in particular for difficult diagnoses in which the classifier can add an additional vote toward the therapeutic choice. For future implementation of our MGCs in the clinical routine, additional health economics analyses and external validation are needed.
